# Supplementary material for: Directing lateral growth of lithium dendrites in micro-compartmented anode arrays for safe lithium metal batteries
Source: Nat Commun. 2018 Jan 31;9:464. doi: 10.1038/s41467-018-02888-8 (PMC5792551; doi:10.1038/s41467-018-02888-8)
Supplement: Supplementary file 1 — Supplementary Information [file 41467_2018_2888_MOESM1_ESM.pdf]

Supplementary Figures

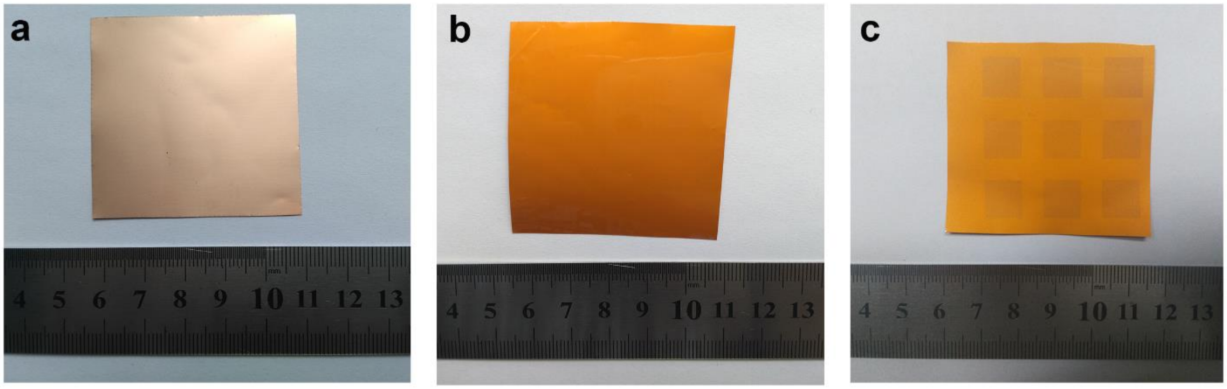

Supplementary Figure 1: Digital images of E-Cu at different preparation status. **a** Commercial Cu foil (pristine). **b** PI film clad Cu foil (after hot lamination). **c** PI film clad Cu foil with interior concave arrays (after laser process).

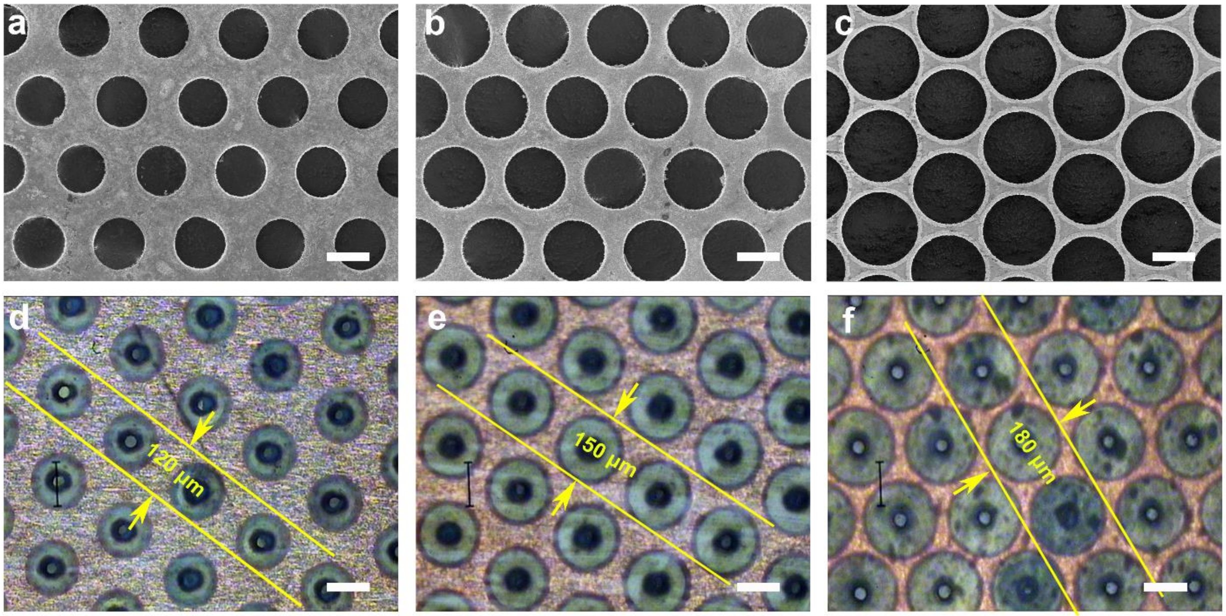

Supplementary Figure 2: Characterization of E-Cu. a-c SEM images and **d-f** optical microscopic images of E-Cu current collector after etching **a, d** 3 h, **b, d** 5 h and **c, f** 7 h, respectively. Scale bars are 100 μm in **a**-**f**.

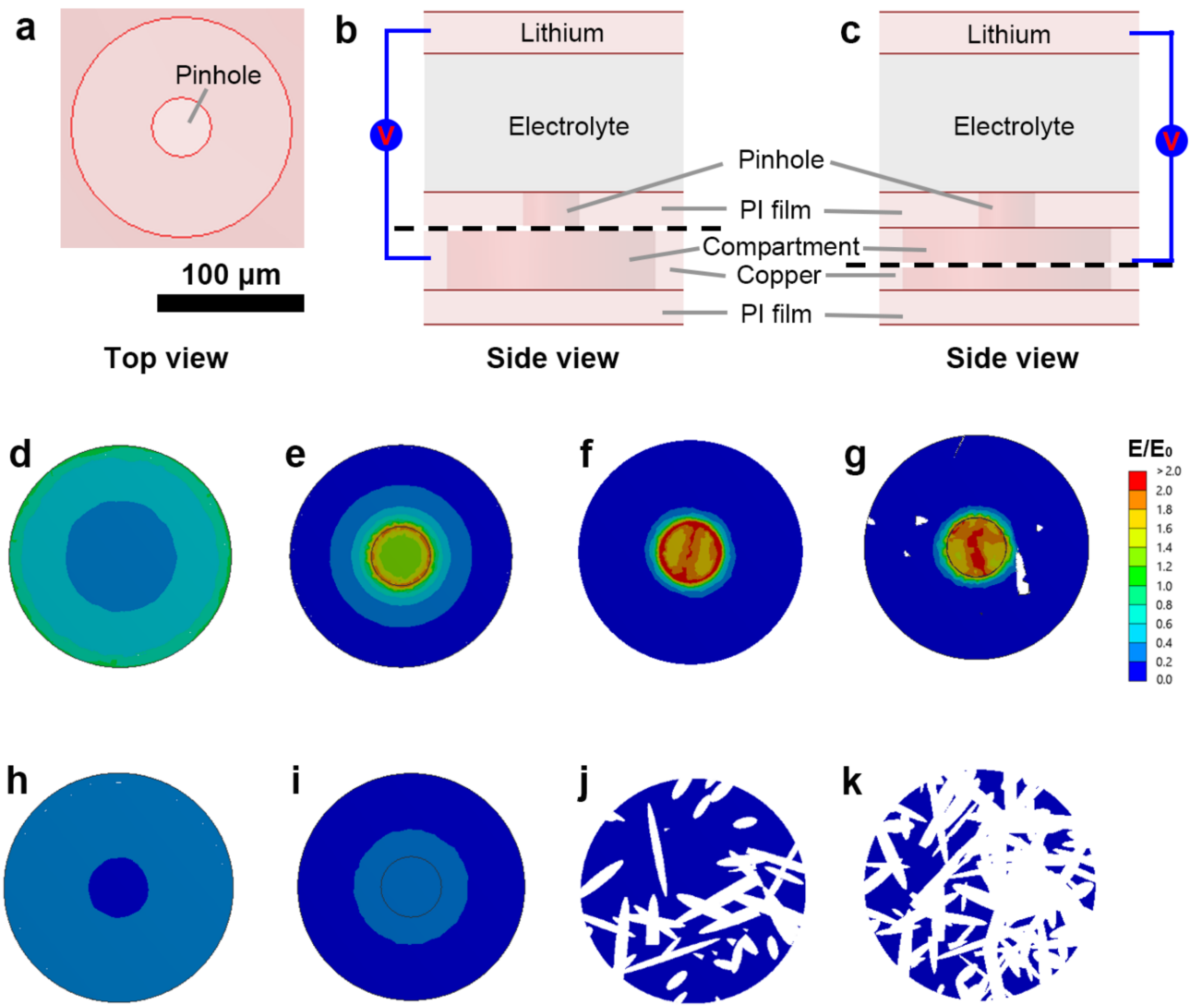

Supplementary Figure 3: Simulation (top view) of the normalized electric field magnitude distribution inside the compartment with different lithium deposition volume %. a Top view of the simulation model. **b-c** Side view of the simulation model. **d-g** Cross-sectional views of the electric field magnitude distribution right under the PI film (thick black dash line in Supplementary Fig. 3b). **h-k** Cross-sectional views of the electric field magnitude distribution at the mid-plane of the compartment (thick black dash line in Supplementary Fig. 3c). The white color parts are Li dendrites within the compartment. For comparison purpose, **d** and **h** are the cases with 0% deposition and with no PI film. **e** and **i** are the cases with 0% deposition. **f** and **j** are the cases with 20% deposition. **g** and **k** are the cases with 50% deposition.

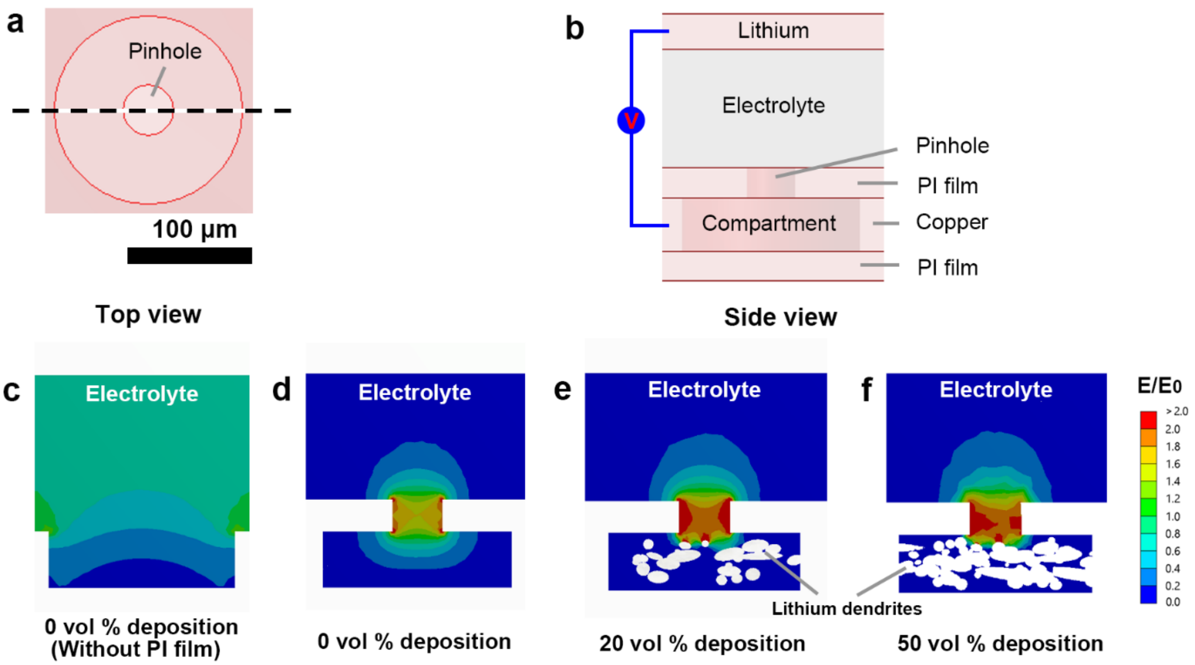

Supplementary Figure 4: Simulation (side view) of the normalized electric field magnitude distribution inside the compartment with different lithium deposition volume %. a Top view of the simulation model. **b** Side view of the simulation model, which locates at the XZ-plane (thick black line in Supplementary Fig. 4a). **e**Cross-sectional views of the electric field magnitude distribution in different simulation cases: **c** is the case with 0% deposition and no PI film for control purpose. **d** is the case with 0% deposition. **e** is the case with 20% deposition. **f** is the case with 50% deposition. These results show that the electric field can still propagate into the compartment with the presence of the PI layer.

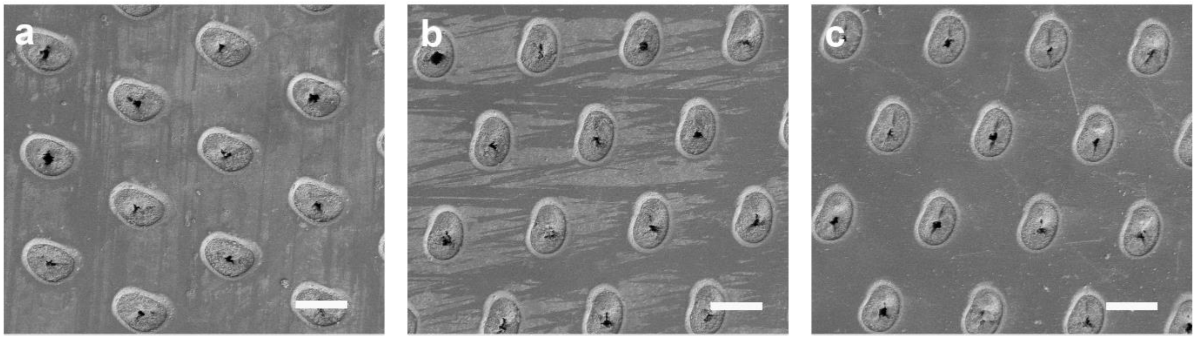

Supplementary Figure 5: Surface top-view images of Li anodes without peeling off the upper PI film after depositing different amounts of Li metal at 0.5 mA cm^{-2}. **a** 0.5 mA h cm^{-2}, **b** 1 mA h cm^{-2} and **c** 2 mA h cm^{-2}. Scale bars in are 100 μm in **a**-**c**.

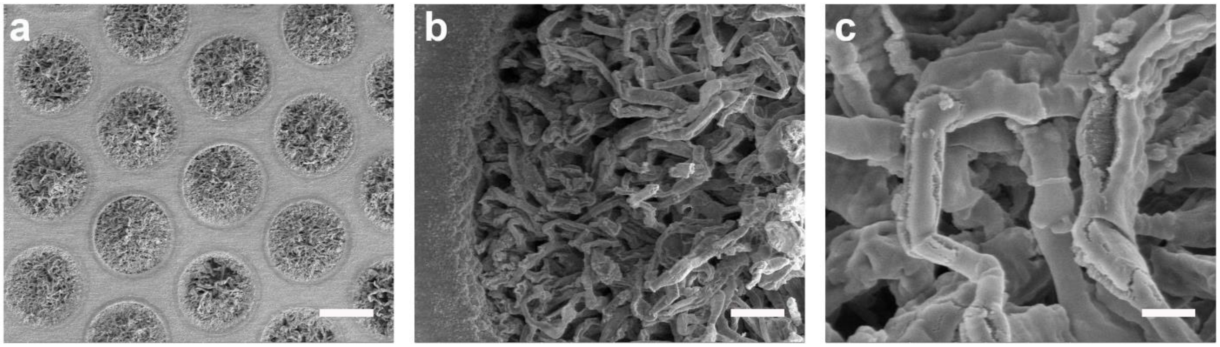

Supplementary Figure 6: Top-view images of E-Cu after depositing 2 mA h cm^{-2} of Li metal at 1.0 mA cm^{-2}. **a** SEM image of E-Cu after peeling off the upper PI film. **b, c** SEM images of E-Cu at higher magnifications. Scale bars are 100 μm, 10 μm and 2 μm in **a**, **b** and **c**, respectively.

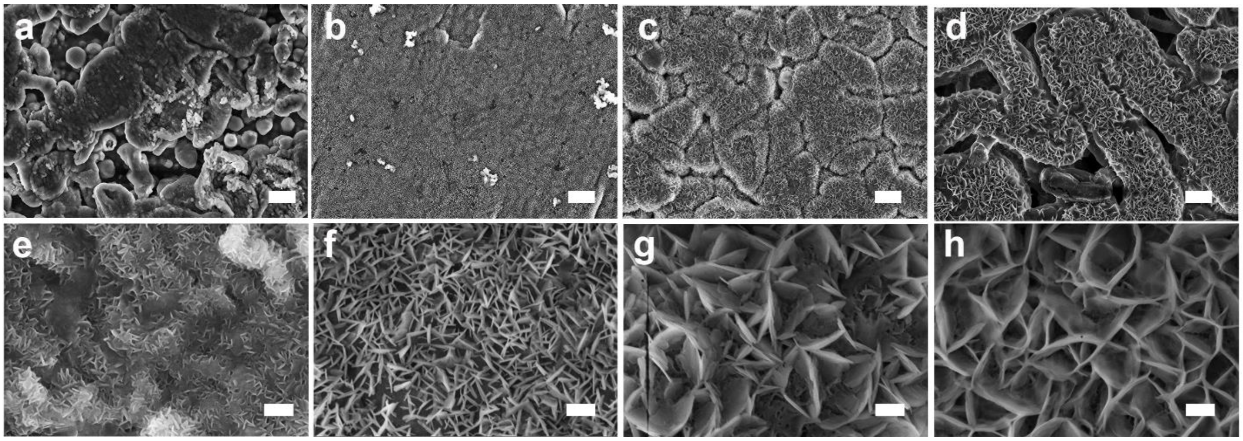

Supplementary Figure 7: SEM images of Li dendrites formed on P-Cu with different amounts of deposited Li metal. a, e 0.5 mA h cm^{-2}, **b, f** 1 mA h cm^{-2}, **c, g** 2 mA h cm^{-2}, and **d, h** 4 mA h cm^{-2}. Scale bars are 2 μm in **a-d** and 200 nm in **e-f**, respectively.

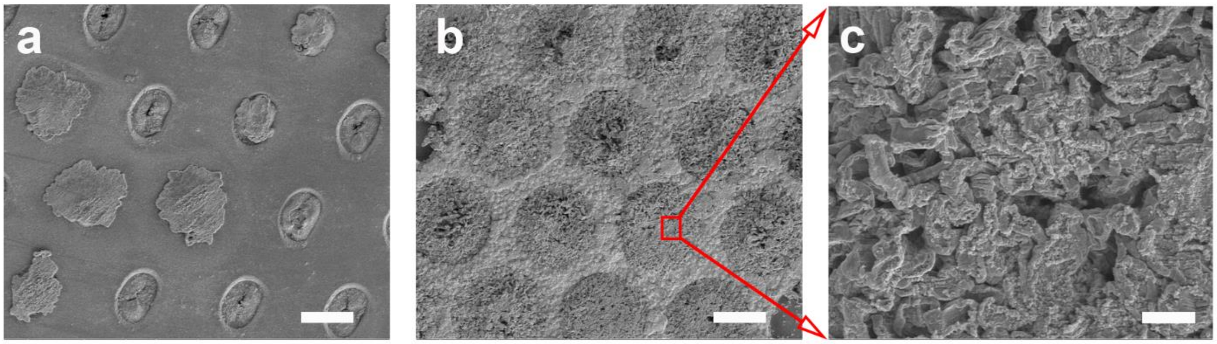

Supplementary Figure 8: SEM images of E-Cu based Li anode with a deposition capacity of 4 mA h cm^{-2}. a Surface top-view image of anode without peeling off PI film. **b** Interior top-view image of anode after peeling off the surface PI film. **c** Magnified image of anode from selected area in Supplementary Figure 8b. Scale bars are 100 μm in **a** and **b**, and 10 μm in **c**.

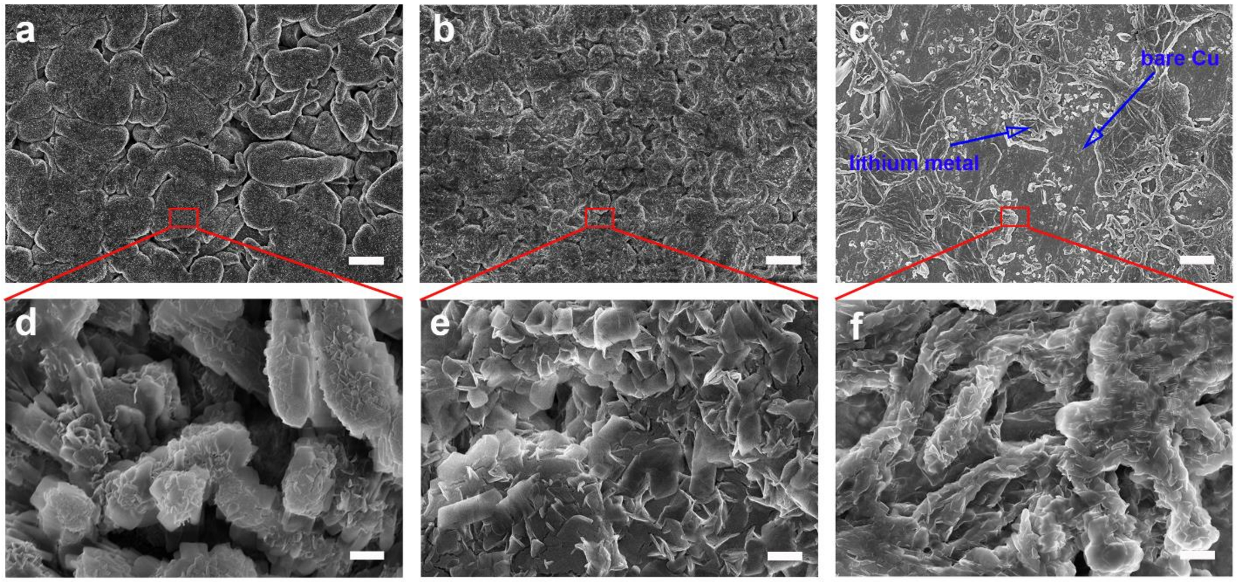

Supplementary Figure 9: Top-view images of P-Cu based anode after lithium stripping. a, d 0.5 mA h cm^{-2}, **b, e** 1.0 mA h cm^{-2}, and **c, f** 2 mA h cm^{-2} of Li metal, before which 2 mA h cm^{-2} of Li metal is deposited into P-Cu firstly. Scale bars are 5 μm in **a-c**, and 500 nm in **d-f**.

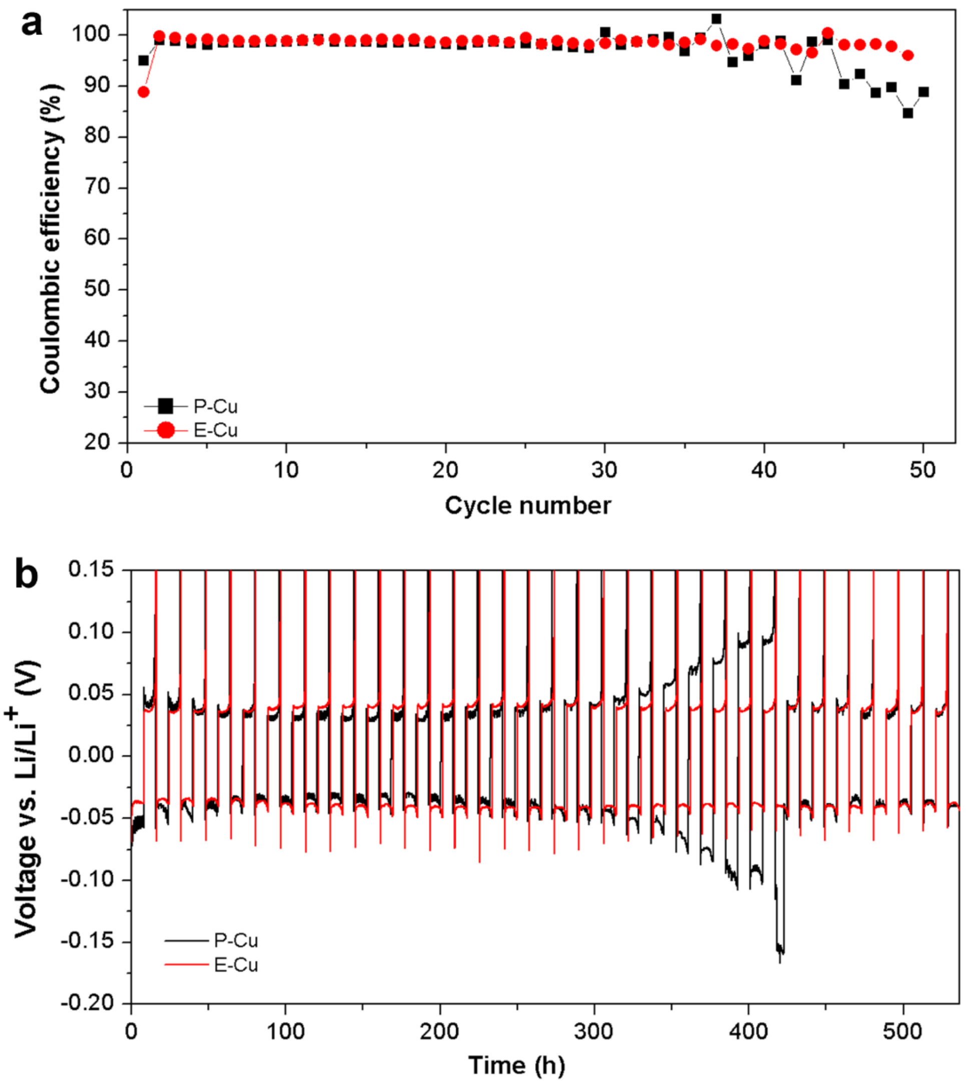

Supplementary Figure 10: Comparison of cycling performances. **a** Coulombic efficiency and **b** voltage-time profile of P-Cu and E-Cu at 0.5 mA cm^{-2} with 4.0 mA h cm^{-2} of plating/stripping lithium metal within 50 cycles.

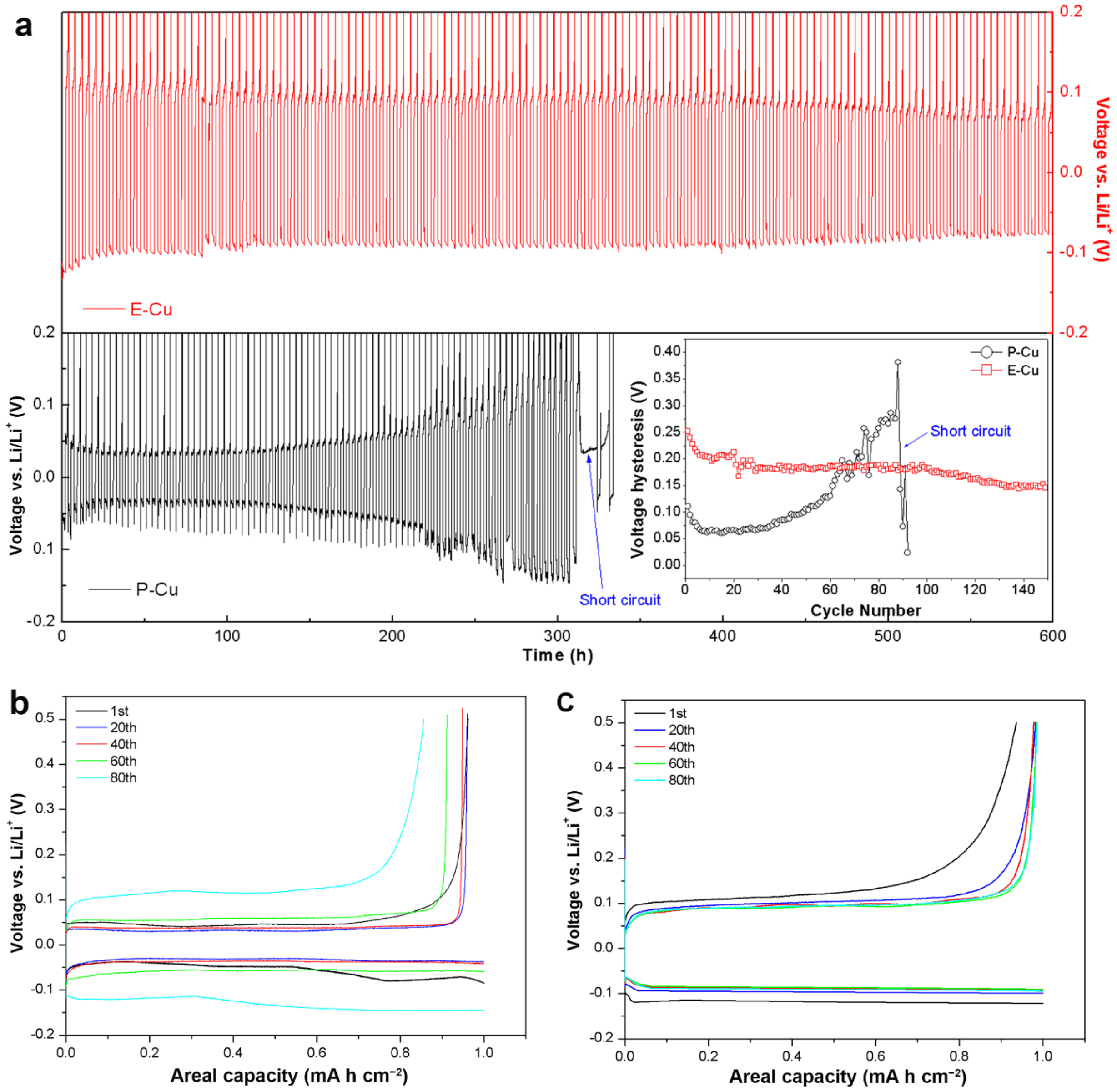

Supplementary Figure 11: Electrochemical performances of Li anodes. a voltage-time profile and average voltage hysteresis (inset) of Li metal anode in Cu@Li//Li systems based on E-Cu and P-Cu with a plating-stripping Li metal of 1.0 mA h cm^{-2} at 0.5 mA cm^{-2}. **b, c** Voltage profiles of Li plating/stripping on **b** P-Cu and **c** 3D E-Cu with a current density of 0.5 mA cm^{-2} after varied charge/discharge cycles. The amount of Li cycled was 1 mA h cm^{-2}.

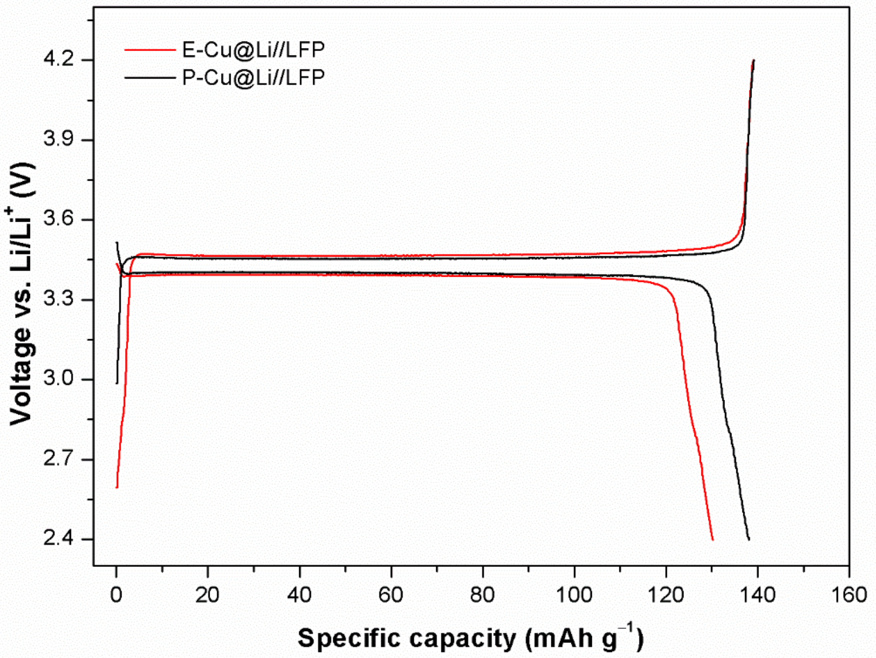

Supplementary Figure 12: Voltage-capacity profile of the initial charging/discharging cycle. Charge and discharge curves of E-Cu@Li//LFP batteries and P-Cu@Li//LFP during the 1st cycle.

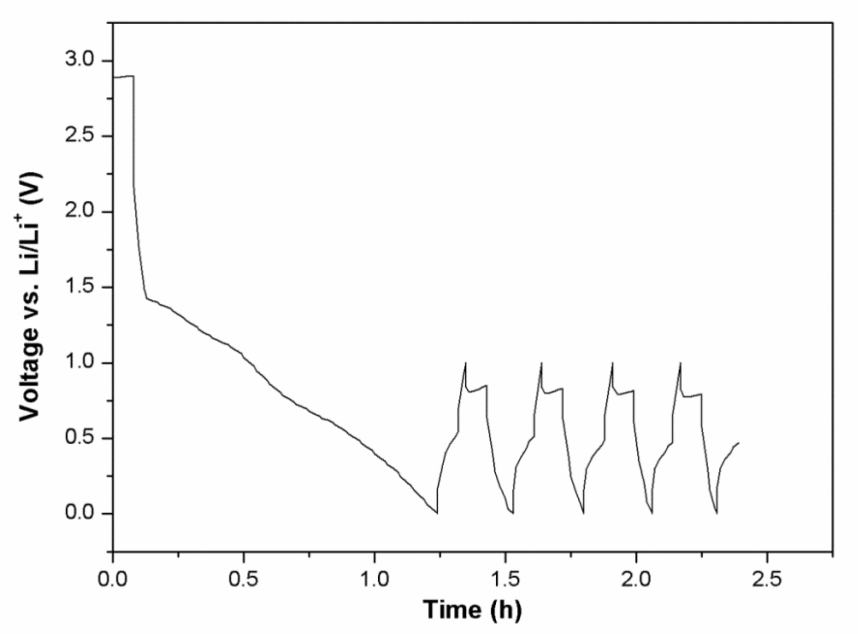

Supplementary Figure 13: Voltage-time profile during activation process. Typical discharging/charging voltage-time profile during initialization process.

Supplementary Tables

Supplementary Table 1. Geometrical parameters and calculated results of E-Cu

Number	Interval distance for two adjacent compartments (μm)	Diameter of compartments (μm)	Electroactive area ratio	Effective pore volume ($cm^3\ cm^{-2}$)	Areal capacity of accommodated Li metal ($mA\ h\ cm^{-2}$)
1	200	200	1.41	~3.53×10^{-3}	7.3
**2***	**200**	**150**	**1.06**	**~1.98×10^{-3}**	**4.1**
3	200	100	0.71	~0.88×10^{-3}	1.8
4	100	100	2.82	~3.53×10^{-3}	7.3
5	100	75	2.12	~1.98×10^{-3}	4.1
6	100	50	1.42	~0.88×10^{-3}	1.8

Supplementary Table 2. Resistivity and Young's modulus of different items for simulation

Item	Resistivity (Ohm m)	Young Modulus (Pa)
Li	1×10^{-5}	4.9×10^{9}
PI film	1×10^{14}	/
Cu	/	2×10^{11}
Electrolyte	1	/
Separator	/	1×10^{9}

Supplementary Discussion

Discussion on the order of magnitude of the diffusion constant

When a sufficient solute concentration is available for the electrochemical reaction at the deposition sites, Chazaviel[5] suggested that the distribution and presence of a locally strong E field considerably promote the growth of metallic dendrites at a velocity of $v_a = \mu_a \cdot E_0$, where μ_a and E_0 are the Li^+ mobility and electric field intensity, respectively. Therefore, we first investigated the order of the magnitude of the Li^+ diffusion constant to determine whether the whole Li^+ concentration flux in the compartment is sufficient to supply for the deposition demand. Taking the electrolyte concentration (*c*) as 10^{20} cm^{-3} (1 M), the typical distance for Li ion diffusion in the vertical direction (*x*) as 10^{-2} cm, and a typical effective current density (*J*) of 0.5 mA cm^{-2}, the diffusion constant (*D*) inside the compartment is calculated to be on the order of 10^{-7} (cm^2 s^{-1}), according to the following equation based on Chazaviel's theory[5, 6, 7]:

$$\frac{\partial c}{\partial x}\Big|_{x=0} = \frac{J\mu_a}{eD(\mu_a+\mu_{Li^+})} \quad \text{(Eq. 1)}$$

Where *c* is the concentration of Li salt, *x* is the diffusion distance of the lithium ions, *J* is the effective current density, *e* is the electronic charge, *D* is the diffusion constant, and μ_a and μ_c are the anionic and Li^+ mobility, respectively.

According to the above theory, when the practical *D* value in our experimental conditions is larger than the order of 10^{-7} (cm^2 s^{-1}), the electric field in the electrodeposition system would become a significant factor towards dendritic lithium growth. From previous literature[8, 9, 10], the Li^+ diffusion constant of 1 M LiTFSI in 1:1 (v/v) DOL:DME has been reported on the order of 10^{-5} (cm^2 s^{-1}). Even considering the dimensional condition that the pinhole area is about 1/10 of the compartment area, the diffusion constant of lithium ion in the pinhole shall be on the order of 10^{-6} (cm^2 s^{-1}), which is still larger than the calculated one from Chazaviel's model (10^{-7}). This indicates that the electrolyte diffusion is on a fast-enough time scale to provide an electrochemically active surface inside the compartment at the range of current densities employed in the experiments (0.25 to 1.0 mA cm^{-2}) and thus can maintain a stable electric current. Therefore, the electric field becomes the dominant factor which determines the growth of lithium dendrite inside the compartments, according to the space-charge model in Chazaviel's theory[5]. Consequently, the influence of the potential gradient generated from the uneven anion depletion during the dynamic charge/discharge process is less significant, and therefore the electrolyte can be assumed to be a static medium (charge carrier) to simplify the simulation.

Simulation of the electric field distribution

Based on the above conclusion, we employed an electrical conduction model[1, 2] to analyze the distribution and strength of the electric field in different simulation cases, and further took these findings as a handle to compare the approximated deposition development of dendrites under different conditions. For clarity, we show the magnitude of the E field as well as its vector distribution, to compare the direction and velocity of dendrite growth. As shown in Fig. 2b, the E field distribution propagates from the top electrode through the pinhole, and laterally extends to the Cu scaffold surface. Consequently, we expect dendrite growth shall initially start from the Cu wall, and then grow towards the pinhole (Supplementary Fig. 3 and 4).

Supplementary Fig. 3 shows the cross-sectional E field under different deposition stage from 0% to 50%. The cross-sectional images are taken from the planes right beneath the top PI film (Supplementary Fig. 3d-g), and at the middle level of the compartment (Supplementary Fig. 3h-k), with both planes parallel to the XY-plane. As seen, all the E field distributions have similar patterns, and start by propagating vertically from the top electrode through the pinhole and then going laterally to the Cu cylindrical electrode. Here, the geometries of the PI and Cu electrode specify this special E field pattern in E-Cu. In the control case (Supplementary Fig. 3d), we take the E field strength on the Cu surface (E_{Cu}) as a reference. Around the cylindrical surface in E-Cu, the E field strength (E_{Cu}*) at the same location in Supplementary Fig. 3e is about 1/2 (near bottom) to 1/7 (near top) that of E_{Cu}. Although E_{Cu}* is generally weaker than E_{Cu}, there is still a sufficient field for the electric current to be established across electrodes and to enable the electrochemical deposition. When comparing the pair cases in Supplementary Fig. 3d and 3e, or the pair cases in Supplementary Fig. 3h and 3i, we can clearly see that the field at the top cross-section is stronger than that at the lower cross-section, indicating that the existence of the insulating PI film exhibits some distortion effect on the E field. However, the E field can still propagate into the compartment through the pinhole. Overall, the PI film does not affect the deposition under our experimental conditions, as confirmed from experimental investigations.

Similarly, around the cylindrical zone in Supplementary Fig. 4, the upper compartment remains electrochemically active due to the presence of E field. Notably, the distribution of E_{Cu}* is very even within the compartment in Supplementary Fig. 4d (before Li deposition), thus promoting an even initial deposition of Li metal, as confirmed in Fig. 4a-c. The lower compartment has a much weaker E field and thus becomes a neutral storage space for the deposited Li metal. Here, the reaction in the lower compartment is weakened by the E field shielding effect from the dendrites in the upper layer; this essentially configures the whole compartment as a regulated "Last-In-First-Out" queue for Li dendrites during plating/stripping process, which is also in accordance to the SEM observations.

Supplementary Methods

Theoretical calculation of the effective volume in E-Cu

The interval distance for two adjacent compartments is accurately controlled to be 200 μm during laser processing, thus the distribution density of compartments in E-Cu is 2500 (50×50) compartments in 1×1 cm×cm. After the alkaline etching treatment, compartments in E-Cu present a cylinder shape and have an average diameter (D) of 150 μm and a height (H) of 45 μm. Then, the effective volume of compartments can be calculated based on the following equations:

$$V_{\text{single compartment}} = 1/4 \cdot \pi \cdot D^2 \cdot H \tag{1}$$

Where V_{single} refers to the volume for one single compartment, D represents the average diameter of compartments and H is the height of cylinder compartment (namely the thickness of copper foil interlayer);

$$V_{\text{total}} = 2500 \cdot V_{\text{single compartment}} \tag{2}$$

Where V_{total} refers to the total volume of compartments in E-Cu with a surface area of 1 cm^2;

$$V_S = V_{\text{total}} / S \tag{3}$$

Where Vs means the pore volume areal density and S is the surface area of E-Cu.

Since Lithium metal has a theoretical specific capacity (E_m) of 3860 mA h g^{-1}, and a density (ρ) of 0.534 g cm^{-3}, the maximum areal mass loading of Li metal (Ms) in E-Cu (stuffing all the interior compartments) is theoretically to be:

$$M_S = Vs \cdot \rho \tag{4}$$

Therefore, the areal capacity of Li metal (Es) accommodated in E-Cu can be obtained as:

$$E_S = E_m \cdot M_S \tag{5}$$

According to the intrinsic parameters of E-Cu, the Vs and Ms are calculated to be ~1.98 $\times 10^{-3}$ cm^3 cm^{-2} and 1.06 mg cm^{-2}, respectively. Thus, the areal capacity density of Li anode based on E-Cu is estimated to be up to 4.1 mA h cm^{-2}.

Similarly, E-Cu with different electroactive area ratio can be obtained by adjusting the processing parameters, such as the interval distance for two adjacent compartments, diameter of compartments and the thickness of the copper interlayer. (Supplementary Table 1)

Simulation setup

Finite element analysis (FEA) simulations were performed to predict the compartment effects on the electric field (E field) distribution as well as the growth of lithium dendrites inside the compartments of E-Cu. The ANSYS models consist of electrical conduction model[1, 2] and structural stress analysis model[3, 4] (based on the Hook's law). In this work, all numerical simulation analysis about E-Cu is conducted on one compartment (Fig. 3, top and side views), which is a typical unit of E-Cu and thus is expected to reflect the average phenomena occurred in all other compartments.

As for bare E-Cu model (see Supplementary Fig. 3b), the compartment has a cylindrical structure with the diameter (D) of 150 μm and height (H) of 45 μm, and the thickness of the upper PI film is 25 μm. The height of the bulk electrolyte in the domain is assumed to be 100 μm. Typical conductivity and mechanical strength are assumed for Li metal, PI film, Cu, electrolyte, and separator, as shown in Supplementary Table 2. The conductivity of Li dendrites is considered to be half of normal Li metal, due to the existence of crystal boundaries in dendrites; even so, the conductivity of Li dendrites is still much better than that of the PI membrane and electrolyte.

Then, we built the E-Cu@Li structure model based on one compartment with randomly distributed cylindrical Li dendrites via the Monte Carlos method with a random generator (see Fig. 3a), where each of these dendrites randomly starts from the Cu scaffold and extents into the compartment. The diameter and length of each cylindrical dendrite are generated as a white noise distribution with the following ranges: according to the SEM observations (Fig. 4), the diameter range of the dendrites is estimated to be from 3 to10 μm, while the length distribution is from 0 to 120 μm. The total number of dendrites depends on the volume of plated lithium in the compartments, which varies from 0% to 60% in the present simulated cases. When a potential difference of V_0 (V) is applied across the top and bottom electrodes, we can obtain an electric field intensity of $E_0 = V_0/100$ (V μm^{-1}) generated across these two electrodes. Then, we can use this E_0 to normalize the values of corresponding E field results in different simulation cases as discussed below (Supplementary Fig. 3 and 4).

It is noted that the electrolyte was assumed as a statically distributed medium instead of a dynamic one when calculating the electric field for all cases. During the simulation of von Mises stress distributions on the dendrite protrusions (Fig. 3), the diameter of the simulated protrusion is assumed to be 5 μm, which is positioned at the center of the pinhole.

Supplementary References

1. Lin, Y. C., Li, M. & Wu, C. C. Simulation and experimental demonstration of the electric field assisted electroporation microchip for in vitro gene delivery enhancement. *Lab Chip* **4**, 104−108 (2004).

2. Aryanfar, A., Brooks, D., Merinov, B. V., Goddard, W. A., III., Colussi, A. J. & Hoffmann, M. R. Dynamics of lithium dendrite growth and inhibition: Pulse charging experiments and monte carlo calculations. *J. Phys. Chem. Lett.* **5**, 1721−1726 (2014).

3. Ge, M., Rong, J., Fang, X. & Zhou, C. Porous doped silicon nanowires for lithium ion battery anode with long cycle life. *Nano Lett.* **12**, 2318−2323 (2012).

4. Greve, L. & Fehrenbach, C. Mechanical testing and macro-mechanical finite element simulation of the deformation, fracture, and short circuit initiation of cylindrical lithium ion battery cells. *J. Power Sources* **214**, 377−385 (2012).

5. Chazalviel, J. N. Electrochemical aspects of the generation of ramified metallic electrodeposits. *Phys. Rev. A* **42**, 7355−7367 (1990).

6. Rosso, M., *et al.* Dendrite short-circuit and fuse effect on li/polymer/li cells. *Electrochimi. Acta* **51**, 5334−5340 (2006).

7. Bruce, P. G. & Vincent, C. A. Steady state current flow in solid binary electrolyte cells. *J. Electroanal. Chem. Interfac.* **225**, 1−17 (1987).

8. Liu, W., Lin, D., Pei, A. & Cui, Y. Stabilizing lithium metal anodes by uniform li-ion flux distribution in nanochannel confinement. *J. Am. Chem. Soc.* **138**, 15443−15450 (2016).

9. Zheng, G., *et al.* Interconnected hollow carbon nanospheres for stable lithium metal anodes. *Nat. Nanotechnol.* **9**, 618−623 (2014).

10. Brissot, C., Rosso, M., Chazalviel, J. N. & Lascaud, S. Dendritic growth mechanisms in lithium/polymer cells. *J. Power Sources* **81–82**, 925−929 (1999).
